# A Novel Electromagnetic Driving System for 5-DOF Manipulation in Intraocular Microsurgery

**DOI:** 10.34133/cbsystems.0083

**Published:** 2024-03-23

**Authors:** Yangyu Liu, Dezhi Song, Guanghao Zhang, Qingyu Bu, Yuanqing Dong, Chengzhi Hu, Chaoyang Shi

**Affiliations:** ^1^ Key Laboratory of Mechanism Theory and Equipment Design of Ministry of Education, School of Mechanical Engineering, Tianjin University, Tianjin 300072, China.; ^2^ Guangdong Provincial Key Laboratory of Human-Augmentation and Rehabilitation Robotics in Universities, Department of Mechanical and Energy Engineering, Southern University of Science and Technology, Shenzhen 518055, China.

## Abstract

This work presents a novel electromagnetic driving system that consists of eight optimized electromagnets arranged in an optimal configuration and employs a control framework based on an active disturbance rejection controller (ADRC) and virtual boundary. The optimal system configuration enhances the system’s compatibility with other ophthalmic surgical instruments, while also improving its capacity to generate magnetic force in the vertical direction. Besides, the optimal electromagnet parameters provide a superior comprehensive performance on magnetic field generation capacity and thermal power. Hence, the presented design achieves a stronger capacity for sustained work. Furthermore, the ADRC controller effectively monitors and further compensates the total disturbance as well as gravity to enhance the system’s robustness. Meanwhile, the implementation of virtual boundaries substantially enhances interactive security via collision avoidance. The magnetic and thermal performance tests have been performed on the electromagnet to verify the design optimization. The proposed electromagnet can generate a superior magnetic field of 2.071 mT at a distance of 65 mm with an applied current of 1 A. Moreover, it demonstrates minimal temperature elevation from room temperature (25 °C) to 46 °C through natural heat dissipation in 3 h, thereby effectively supporting prolonged magnetic manipulation of intraocular microsurgery. Furthermore, trajectory tracking experiments with disturbances have been performed in a liquid environment similar to the practical ophthalmic surgery scenarios, to verify the robustness and security of the presented control framework. The maximum root mean square (RMS) error of performance tests in different operation modes remains 35.8 μm, providing stable support for intraocular microsurgery.

## Introduction

**Fig. 1.**
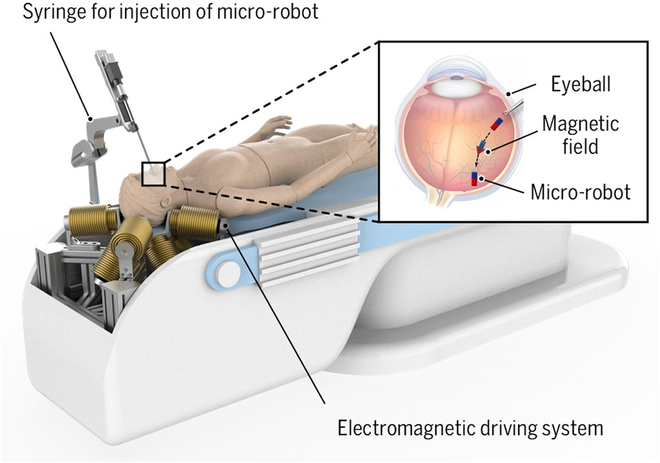
Illustration of an electromagnetic driving system to manipulate a micro-robot within the patient’s posterior eye.

Intraocular microsurgery has witnessed a transition from the utilization of conventional handheld surgical tools to the adoption of robot-assisted surgery, owing to its ability to effectively mitigate the surgeons’ physiological tremors during procedures and achieve precise motion scaling. Consequently, this advancement significantly enhances the accuracy and stability of surgeries [1–3]. The robot-assisted systems commonly employ a remote center of motion mechanism to manipulate conventional vitreoretinal tools, which are introduced into the patient’s anterior eye through the sclerotomy [4]. However, with increasing proximity to the posterior eye, robot-assisted devices may position the instruments too deeply or exert excessive scleral forces under the surgeon’s inadvertent control, which can traumatize the retina or sclera and result in hemorrhages or even severe injury. The above causes have led to the incidence of intraoperative and postoperative complications ranging from 2% to 30% [5]. The electromagnetic driving systems are proposed for the flexible 5-DOF (degree of freedom) magnetic manipulation of a micro-robot within the posterior eye, enabling precise targeted drug delivery [6–12], as illustrated in Fig. 1. Meanwhile, these 5-DOF systems pose a distinct actuation paradigm compared with the existing robotic-assisted systems. It typically employs a force-controlled mode rather than a position-controlled mode, which makes the micro-robot a safer instrument for interacting within the posterior eye. In force-controlled mode, the electromagnetic driving system can effectively mitigate the risk of causing irreparable retinal damage by imposing limits on interacting forces, even in situations involving patient movement or system failure [13]. However, it is challenging to generate high-intensity magnetic fields and magnetic forces within a large workspace [9]. Therefore, the design optimization of the system configuration and electromagnet parameters for providing a high magnetic field and magnetic force generation capacity has been emerging and attracting broad attention.

The design optimization of the system configuration commonly involves the arrangement of eight electromagnets, and the arrangement options primarily comprise square antiprism [14,15], square prism [16–19], open asymmetric [20], and OctoMag style [13,21]. The first two types of configurations have been commonly utilized in the literature and in several large-scale commercial systems. However, these configurations restrict surgeons’ access to additional instruments for the anterior eye injection of micro-robots during surgical procedures. The third type of configuration offers enhanced accessibility to the manipulation workspace, but it exhibits limited capability in generating magnetic force [8,20]. The configuration similar to the OctoMag style represents the pioneering design for intraocular microsurgery (e.g., vitreoretinal surgery) and is widely acknowledged as suitable for intraocular treatment [13,22–24]. It has been optimized during the design process, considering the magnetic force generation capacity. However, the optimization process overlooks the particular impact of magnetic force generation capacity in the vertical direction, which plays a crucial role in intraocular micro-robot manipulation. The insufficient force generation capacity in the vertical direction may result in additional power consumption during prolonged operation, thereby impacting the sustained surgical durations of the system. Thus, developing an optimization process for the system configuration is crucial to consider a wider range of factors, enhancing the vertical force generation capacity while ensuring compatibility with other instruments.

The electromagnet parameters are also a crucial component of the system design, significantly determining the magnetic field generation capacity. The parametric design conducted by Sun and colleagues [25,26] aimed to characterize the influence of iron core parameters on the generated magnetic field while neglecting the coil parameters. The design optimization process of the MiniMag system evaluated the impact of coil slope angle on electromagnet performance and space compatibility [21]. However, it neglects to analyze the thermal power of the electromagnet, which is a crucial aspect for ensuring the reliable operation of such electromagnets. The BatMag system incorporates a water-cooling mechanism to optimize thermal management [27]. However, it increases the size of the system and may cause interference between electromagnets. Parameterizing the electromagnets to optimize thermal management may present a more desirable approach. The optimization process of the Advanced Robotics for Magnetic Manipulation (ARMM) system provides the electromagnet geometry that maximizes the magnetic field for a given mass and heat dissipation constraints [28]. Nevertheless, it does not optimize the thermal power of the electromagnet. Besides, all the aforementioned optimizations were analyzed under fixed driving current conditions, potentially limiting their ability to demonstrate consistent optimal performance across varying driving current values. Therefore, a multi-objective optimization process excluding the effect of current should be proposed to enhance the magnetic field generation capacity of the electromagnets while constraining the thermal power.

To tackle the abovementioned issues, this work presents a novel electromagnetic driving system for 5-DOF magnetic manipulation in intraocular microsurgery. Two-step design optimization attempting to obtain optimal system configuration and electromagnet parameters have been presented and implemented to enhance the capacity for sustained work. With the proposed configuration optimization procedure and the multi-objective optimization of the electromagnets, the system can perform a more precise and stable manipulation and has obtained a stronger capacity for sustained work. In addition, the system utilizes a control framework incorporating the active disturbance rejection controller (ADRC) and virtual boundary to enhance robustness and security in intraocular microsurgery. Simulation and analysis have been performed to evaluate the influences of the proposed design optimization and control framework. The performance evaluation and trajectory tracking performance tests in different operation modes are implemented with the presented novel electromagnetic driving system and validate its performances and effectiveness.

## Materials and Methods

### Design of the presented electromagnetic driving system

#### Design and working principle of the presented electromagnetic driving system

**Fig. 2.**
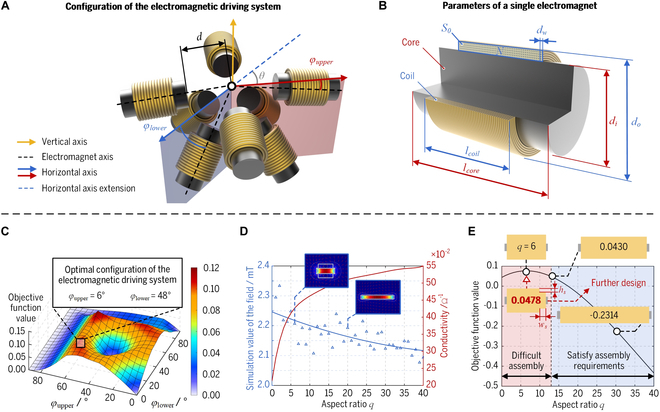
Two-step design optimization of the proposed 5-DOF electromagnetic driving system. (A) Initial system configuration and parameters to be optimized. (B) Parameters of the coil and core for a single electromagnet. (C) Relationship between the configuration parameters and the objective function value. (D) FEM-based simulation values of magnetic flux density and conductivity for a given aspect ratio. (E) Comparison of objective function values for different electromagnet parameters.

A 5-DOF electromagnetic driving system consisting of eight optimized electromagnets arranged in an optimal configuration has been proposed in this work, as depicted in Fig. 2A. The electromagnets are oriented toward the center point of the workspace and positioned at a distance *d* (*d* = 65 mm) from the center. The distance *d* is designed to provide an open volume between the electromagnets for accommodating a small animal head, and the usable workspace within the open volume necessitates covering the interior volume of the subject’s eye. In addition, the electromagnets are categorized into two upper and lower sets, with each consisting of four electromagnets. The angle between the groups *θ* is set to 45° to avoid potential collisions between the electromagnets, and both groups are uniformly distributed around a vertical axis. Meanwhile, *φ*_upper_ and *φ*_lower_ are defined as the angle between the electromagnet axis and the corresponding horizontal axis, as shown in Fig. 2A. With driving currents of 0 to 8 A, this system can generate a maximum magnetic field of approximately 40 mT within the usable workspace. By regulating the driving current of each electromagnet, the desired magnetic field and the corresponding field gradient can be obtained to wirelessly apply the magnetic torque and force on the micro-robot. Hence, the micro-robot inside the magnetic workspace is capable of producing 3-DOF translation and 2-DOF rotation motions under the system manipulation, except for rotating around its axis of the magnetic moment.

The magnetic force ***F***_***m***_ ∈ ℝ^3^ and torque ***τ***_***m***_ ∈ ℝ^3^ on the micro-robot can be respectively calculated as:

$$F_{m}\left(P\right)=\nabla \left(M∙B\left(P\right)\right)$$ (1)

$$\tau_{m}\left(P\right)=M\times B\left(P\right)$$ (2)

The micro-robot is regarded as a permanent magnet at the point ***P*** ∈ ℝ^3^ in the workspace, and its magnetic moment is represented by employing vector ***M*** ∈ ℝ^3^ with a constant magnitude ‖***M***‖. Meanwhile, ***B***(***P***) ∈ ℝ^3^ represents the magnetic field vector at the location of the micro-robot and ∇ denotes the Nabla operator. The magnetic field and field gradient generated by the entire system can be approximated as a linear superposition of those from individual electromagnets [13]. Besides, the linear relationship between the current and the magnetic field or field gradient can be observed when the core (made of DT4 material) has not yet reached magnetic saturation *B_s_* (i.e., ‖***B*****(*****P*****)**‖ < *B_s_*). In the premises of a quasi-static field, the magnetic field and field gradient generated by the entire electromagnetic driving system can be decomposed into matrix multiplication. They can be expressed as follows:

$$B\left(P\right)=\sum_{i=1}^{n}{\tilde{B}}_{i}\left(P\right)I_{i}=\tilde{B}\left(P\right)I$$ (3)

$$\begin{array}{c} \left[\begin{array}{c} \frac{\partial B\left(P\right)}{\partial x}\\ \frac{\partial B\left(P\right)}{\partial y}\\ \underset{\;}{\frac{\partial B\left(P\right)}{\partial z}} \end{array}\right]=\sum_{i=1}^{n}\left[\begin{array}{c} \frac{\partial {\tilde{B}}_{i}\left(P\right)}{\partial x}\\ \frac{\partial {\tilde{B}}_{i}\left(P\right)}{\partial y}\\ \underset{\;}{\frac{\partial {\tilde{B}}_{i}\left(P\right)}{\partial z}} \end{array}\right]I_{i}=\left[\begin{array}{c} \frac{\partial \tilde{B}\left(P\right)}{\partial x}\\ \frac{\partial \tilde{B}\left(P\right)}{\partial y}\\ \underset{\;}{\frac{\partial \tilde{B}\left(P\right)}{\partial z}} \end{array}\right]\;I=\frac{\partial \tilde{B}\left(P\right)}{\partial P}I \end{array}$$ (4)

where *n* denotes the number of electromagnets (in the proposed system 5-DOF magnetic manipulation, *n* = 8 ). $\tilde{B}\left(P\right)\in {\mathbb{R}}^{3\times 8}$ and $\frac{\partial \tilde{B}\left(P\right)}{\partial P}\in {\mathbb{R}}^{9\times 8}$ represent the matrix of magnetic field and field gradient, respectively. ***I*** ∈ ℝ^8^ represents the driving current vector of the eight optimized electromagnets in the system. The utilization of magnetic field control is commonly preferred over torque control in the majority of scenarios of magnetic manipulation. Hence, ***B***(***P***) and ***F***_***m***_ can be derived by combining Eqs. 1 to 4:

$$\begin{array}{c} \left[\begin{array}{c} B\left(P\right)\\ F_{m}\left(P\right) \end{array}\right]=\left[\begin{array}{c} \;\begin{array}{c} \tilde{B}\left(P\right) \end{array}\\ \begin{array}{c} M^{T}\underset{\;}{\frac{\partial \tilde{B}\left(P\right)}{\partial x}} \end{array}\\ \begin{array}{c} M^{T}\frac{\partial \tilde{B}\left(P\right)}{\partial y}\\ M^{T}\underset{\;}{\frac{\partial \tilde{B}\left(P\right)}{\partial z}} \end{array} \end{array}\right]\left[\begin{array}{c} I_{1}\\ \underset{\;}{\vdots }\\ I_{8} \end{array}\right]=A\left(M\;,\;P\right)\;I \end{array}$$ (5)

where ***A***(***M***, ***P***) ∈ ℝ^6 × 8^ is defined as the actuation matrix of the electromagnetic driving system. This matrix can be utilized to analyze the effect of configuration on the performance of the electromagnetic driving system.

#### Configuration optimization for enhancing magnetic force generation capability

Configuration optimization has been applied to enhance the magnetic force generation capability of the proposed electromagnetic driving system. For a given electromagnet configuration, the minimal singular value *σ*_min_ can be utilized as a quantitative measure of force generation capacity in the worst-case scenario and serves as the objective function [13,29]. Furthermore, the restrictive conditions on the angle *φ*_upper_ and *φ*_lower_ are incorporated during optimization processes to allocate space above the workspace, facilitating compatibility with other ophthalmic surgical instruments. In summary, the configuration optimization can be expressed as:

$$\text{max}\;\sigma_{\text{min}}\left(\varphi_{\text{upper}},\;\varphi_{\text{lower}}\right)$$ (6)

subject to:

$$\left\{\begin{array}{c} \varphi_{\text{lower}}\in \;\left[0^{{}^{\circ}},\;90^{{}^{\circ}}\right]\\ \varphi_{\text{upper}}\in \;\left[0^{{}^{\circ}},\;90^{{}^{\circ}}\right] \end{array}\right.$$ (7)

where *σ*_min_ denotes the singular value under the worst-case scenario. The worst-case scenario is defined as the situation in which the micro-robot experiences the minimum magnetic driving force, determined by the robot’s own orientation (i.e., the magnetic moment ***M***) and the distribution of field gradient generated at the robot’s position $\left(\text{i.e., the field gradient}\;\frac{\partial B}{\partial P}\right)$. Since the space field gradient is generated by the unit current during this optimization process, its distribution is determined only by the configuration of the electromagnetic drive system. For a given configuration, the driving force applied to the micro-robot is only related to the robot’s orientation. Hence, it is necessary to consider different robot orientations for a specific electromagnetic driving system configuration. If the system can generate an adequate driving force under the worst-case scenario, it can similarly magnetically manipulate the micro-robot with improved maneuverability under other scenarios. Therefore, it is reasonable and necessary for the optimization process to consider different scenarios and take the worst case as the optimization objective. The incorporation of this special consideration ensures that the system can effectively manipulate the micro-robot with different orientations by applying an adequate magnetic force, thereby meeting the surgical requirements and enhancing manipulation flexibility. For a given electromagnet configuration, *σ*_min_ can be obtained by performing singular value decomposition (SVD) calculations on a set of modified actuation matrix $A_{i}^{\prime }\left(M_{i},\;P\right)$, as illustrated below:

$$\begin{array}{c} \left\{\begin{array}{c} \sigma_{\text{min}}=\text{min}\left(\sigma_{1}\;\cdots \;\sigma_{i}\;\cdots \;\sigma_{n}\right)\\ A_{i}^{\prime }\left(M_{i},\;P\right)=U_{i}\Sigma_{i}V_{i}^{T} \end{array}\right.,\;i=1,\;2,\;3,\;\cdots \;,\;n \end{array}$$ (8)

where *σ_i_* denoting the singular values can be obtained from the singular-value matrixes ***Σ***_***i***_. ***U***_***i***_ and ***V***_***i***_ indicate the orthonormal matrixes, which are additions to the SVD calculation result. The optimization routine considers magnetic moment vectors ***M***_***i***_ (representing the micro-robot’s orientation) at different cardinal orientations to ensure that the system possesses isotropic force control authority, as demonstrated in Eq. 8.

Meanwhile, the micro-robot is always located at ***P ***= [0 0 0]^T^ during the optimization process, since the magnetic field and field gradient exhibit minor variation across the small usable workspace. Moreover, two factors *α* and *k_z_* are utilized to modify the actuation matrix, as shown below.

$$A_{i}^{\prime }\left(M_{i\;},\;P\right)=\left[\begin{array}{c} \begin{array}{c} \alpha \cdot \tilde{B}\left(P\right) \end{array}\\ \begin{array}{c} M_{i}^{T}\frac{\partial \tilde{B}\left(P\right)}{\partial x}\\ M_{i}^{T}\frac{\partial \tilde{B}\left(P\right)}{\partial y}\\ \frac{1}{k_{z}}\cdot M_{i}^{T}\frac{\partial \tilde{B}\left(P\right)}{\partial z} \end{array} \end{array}\right]=U_{i}\Sigma_{i}V_{i}^{T},\text{for}\left\{\begin{array}{c} 0<\alpha \ll 1\\ k_{z}=\frac{F_{g}+F_{r}-F_{b}}{F_{r}} \end{array}\right.$$ (9)

As *α* approaches zero, the matrix $A_{i}^{\prime }\left(M_{i}\;,\;P\right)$ excludes the effects of magnetic fields and provides information on magnetic forces only. Besides, the coefficient *k_z_* serves as a compensatory factor employed to rectify the matrix ***A***(***M***, ***P***). This utilized coefficient can increase the weight of the vertical force generation capacity of the system during the configuration optimization. Hence, this special consideration enables the proposed system to compensate for the gravity of the micro-robot with less energy consumption. The factor *k_z_* is defined by the gravitational force *F_g_* of the micro-robot, the resistance *F_r_* exerted by the surrounding liquid, and the buoyancy force *F_b_*. The values of the three forces mentioned above can be obtained by modeling.

The initial configuration has been proposed to simplify the optimization parameters, as depicted in Fig. 2A. The optimization routine attempts to obtain these optimal configuration parameters *φ*_upper_ and *φ*_lower_ by utilizing the ga function from MATLAB 2022b (MathWorks, Natick, MA, USA), as illustrated in Eq. 6. Through the above calculation and optimization, *φ*_upper_ and *φ*_lower_ are determined as 6° and 48°, respectively, as depicted in Fig. 2C.

### Design of a single electromagnet considering magnetic field, thermal power, and platform volume

The optimization of parameters has been implemented to enhance the magnetic field generation capacity of the electromagnets while constraining both the thermal power and the platform volume. This particular consideration significantly diminishes the system’s heat dissipation requirements and prolongs its operational duration. Besides, the consideration of platform volume is imperative due to the surgical space limitation and the necessity for seamless coordination across various surgical areas. Therefore, the objective function *f* can be given as the sum of the magnetic field-to-thermal power ratio (denoted as *q_bh_*) and platform volume *V*. Moreover, the electromagnet parameters mainly consisting of two parts (the coil parameters and the iron core parameters) are summarized in Fig. 2B, and the initialization of electromagnet parameters has been defined based on the design requirements to streamline the optimization routine. In summary, the optimization of parameters can be expressed as:

$$\text{max}\;f\left(q\right)=w_{1}q_{\mathrm{bh}}\left(q\right)-w_{2}V\left(q\right)$$ (10)

subject to:

$$\left\{\begin{array}{c} \begin{array}{c} d_{w}=1.6\;\mathrm{mm}\\ \;N=1,060 \end{array}\\ \begin{array}{c} \;d_{i}=40\;\mathrm{mm}\\ \;l_{\text{core}}=l_{\text{coil}}+25\;\mathrm{mm} \end{array}\\ \;q=\frac{2l_{\text{coil}}}{d_{o}-20\;\mathrm{mm}} \end{array}\right.$$ (11)

where *w*_1_ and *w*_2_ denote weight coefficients. The ratio *q_bh_* is employed as a means to represent the magnetic field (*B_i_* ∝ *I_i_*) and thermal power (${\overset{\cdot }{Q}}_{i}\propto I_{i}^{2}$) of an individual electromagnet while excluding the influence of the current magnitude |*I_i_*|. The increase in *q_bh_* enables the system to generate a higher magnetic field with limited thermal power, thereby augmenting magnetic manipulation capability and prolonging surgical durations. The ratio *q_bh_* and platform volume *V* can be roughly calculated employing simplified models as follows:

$$\left\{\begin{array}{c} \begin{array}{c} \;V=\frac{2\pi }{3}\cdot {\left(l_{\text{core}}+65\;\mathrm{mm}\right)}^{3}\\ q_{\mathrm{bh}}=\frac{B_{i}^{2}}{{\overset{\cdot }{Q}}_{i}}=\frac{2k_{c}^{2}\mu_{0}^{2}}{\rho }\cdot \frac{N{\left(d_{i}+d_{o}\right)}^{3}d_{w}^{2}}{{\left[{\left(d_{i}+d_{o}\right)}^{2}+4{\left(l_{\text{core}}+130\;\mathrm{mm}\right)}^{2}\right]}^{3}} \end{array}\\ \begin{array}{c} \;B_{i}=2\mu_{0}k_{c}I_{i}\cdot \frac{N{\left(d_{i}+d_{o}\right)}^{2}}{{\left[{\left(d_{i}+d_{o}\right)}^{2}+4{\left(l_{\text{core}}+130\;\mathrm{mm}\right)}^{2}\right]}^{\frac{3}{2}}}\\ {\overset{\cdot }{Q}}_{i}=I_{i}^{2}R_{i}=2\rho I_{i}^{2}\cdot \frac{N\left(d_{i}+d_{o}\right)}{d_{w}^{2}} \end{array} \end{array}\right.$$ (12)

where *k_c_* denotes the magnification of the magnetic field due to the presence of the iron core. μ_0_ and *ρ* represent the permeability of vacuum and resistivity of wire, respectively. In addition to the aforementioned constant parameters, Eq. 12 also incorporates electromagnet parameters.

The coil parameters (wraps *N*, wire diameter *d_w_*, coil length *l*_coil_, and coil diameter *d_o_*) and the iron core parameters (core length *l*_core_ and core diameter *d_i_*) are summarized in Fig. 2B. The core diameter *d_i_* is chosen as 40 mm to avoid insufficient capacity to generate magnetic fields, and the core length *l*_core_ is 12.5 mm longer than the coil at both ends for easier assembly. In addition, the wraps *N* and wire diameter *d_w_* require particular attention. They hardly affect the magnetic field-to-thermal power ratio *q_bh_*, when the cross-sectional area of the coil ($S_{0}={\mathrm{Nk}}_{w}d_{w}^{2}$) remains constant, as demonstrated in Eq. 12. Here, *k_w_* denotes the error coefficient resulting from the nonuniform winding of the coil and other related factors. The aforementioned conclusion implies that the superior comprehensive performance of a single electromagnet can be achieved through the combination of a smaller *d_w_* accompanied by larger *N*, as well as a larger *d_w_* coupled with fewer *N*. However, a reduced *N* facilitates enhanced resolution of the magnetic field to the current, thereby facilitating more precise regulation of the magnetic field magnitude via the driving current. Therefore, a *d_w_* of maximum size (*d_w_* = 1.6 mm) is selected to maximize the resolution of the magnetic field to the current. The wraps *N* are designed with reference to other platforms (*N* = 1,060), thereby guaranteeing that the proposed electromagnet can possess the capacity to generate a sufficient magnetic field. At this stage, the aspect ratio *q* is the only parameter to be optimized, as demonstrated in Eqs. 10 and 11.

A parametric model of the electromagnet is developed utilizing COMSOL 6.1 software (COMSOL, Burlington, VT, USA) and linked with MATLAB 2022b. The magnetic field magnitude and conductivity for a given aspect ratio *q* can be obtained by solving the model, as shown in Fig. 2D. The optimization results are obtained by normalizing the magnetic field-to-thermal power ratio and platform volume, as shown in Fig. 2E. They indicate that the objective function reaches its maximum value when the aspect ratio is around 6. However, the aspect ratio equal to 6 is situated within a region that suggests the potential for interference, which may lead to difficulties in assembly. Therefore, a further design for assembly convenience is proposed, realized by a stepped shaft with a width *w_s_* of 25 mm and a length *h_s_* of 13 mm at a constant electromagnet cross-sectional area. The finite element modeling (FEM)-based simulation has been employed to compare the magnetic field and field gradient generation capabilities of electromagnets with and without optimization. The simulation results demonstrate relatively limited improvement in terms of magnetic torque generation capacity but exhibit an advantage of 15.5% in magnetic force generation capacity. Nevertheless, the optimized electromagnet still exhibits a more pronounced advantage in terms of overall performance considering magnetic field generation capacity, thermal power, and platform volume, as depicted in Fig. 2E. Meanwhile, it increases the assembly margin twice compared to a regular electromagnet that fulfills assembly specifications. The optimized parameters of the proposed electromagnet are summarized in Table 1.

**Table 1.** Optimized parameters of a single electromagnet

Symbol	Quantity	Value
*N*	Wraps	1,060
*d_w_*	Wire diameter	1.6 mm
*l*_coil_	Coil length	140 mm
*l*_core_	Core length	165 mm
*d_o_*	Coil diameter	92 mm
*d_i_*	Core diameter	40 mm
*h_s_*	Step height	13 mm
*w_s_*	Step width	25 mm

### The proposed ADRC controller

**Fig. 3.**
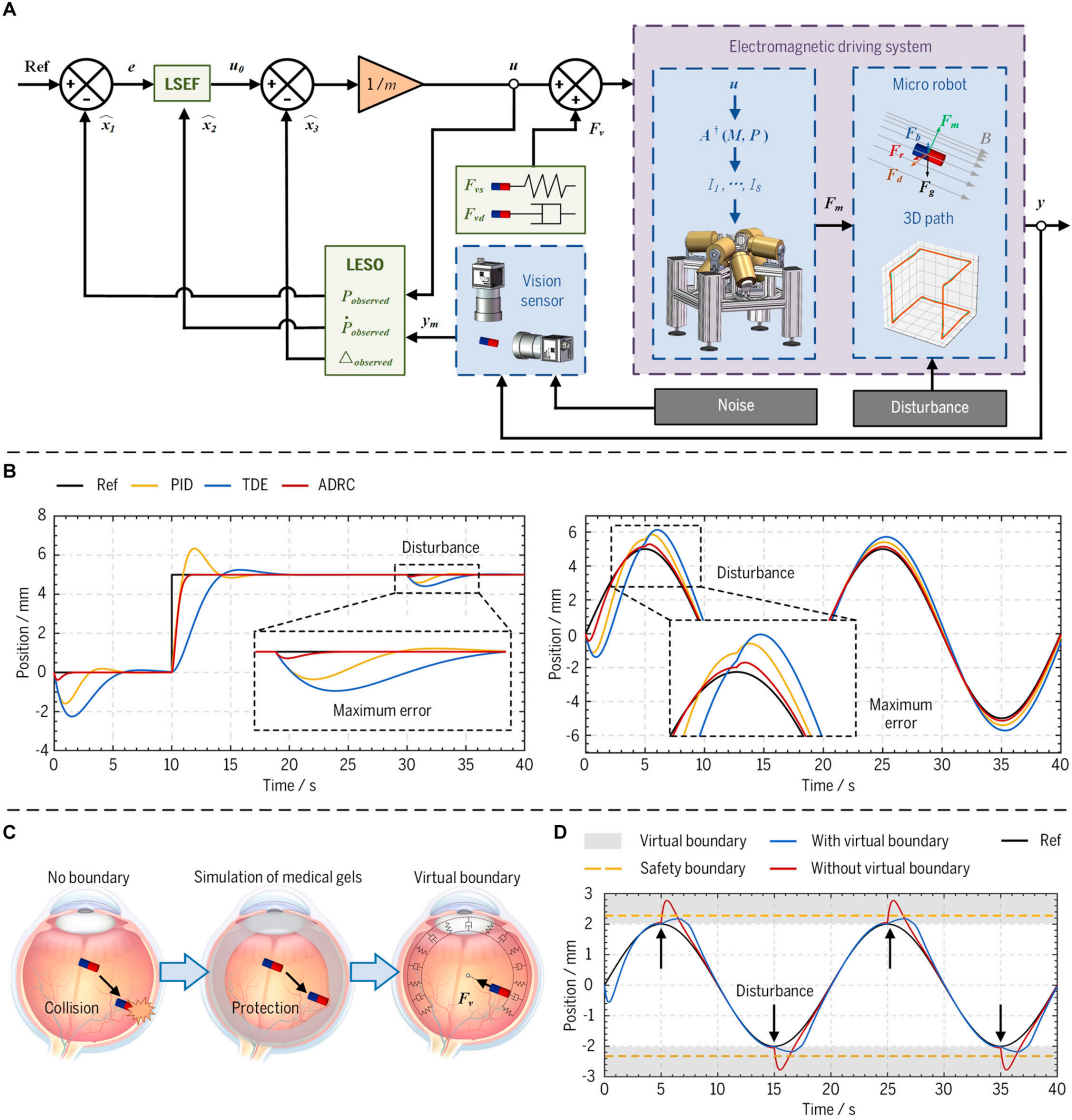
(A) Proposed control framework based on ADRC and virtual boundary. (B) Simulation results for step and sine wave response of the PID, TDE, and ADRC controllers. (C) Illustration of the virtual boundary. (D) Security analysis without and with the virtual boundary.

A model-free adaptive controller incorporating the ADRC-based algorithm has been proposed to enhance the robustness capability of the electromagnetic system, as illustrated in Fig. 3A. The proposed ADRC controller can observe and further eliminate total disturbance, which represents all external forces applied to the micro-robot other than the input magnetic force. Thus, the associated uncertainties and nonlinearities of the system can be effectively mitigated, thereby enhancing the robustness capability of the system. Compared to other works [30,31], this work considers the performance of the ADRC controller in gravity compensation, further ensuring precise and stable magnetic manipulation in liquid environments similar to practical ophthalmic surgery scenarios. The proposed ADRC controller can be defined as:

$$m\cdot {\ddot{p}}_{i}=u+\;△\left(i=x,\;y,\;z\right)$$ (13)

$$u=u_{0}\;-{△}_{\mathrm{ob}}$$ (14)

where *m* represents the mass of the micro-robot and *p_i_* denotes any positional coordinate within the Cartesian coordinate system. The total disturbance is defined as △ = *F_g_* + *F_b_* + *F_r_* + *F_d_*, and *F_d_* represents the disturbances from multiple uncertainties. The disturbances *F_d_* has taken various factors into account, including environmental disturbances, measurement noises, errors in the magnetic field–current model, and other related factors. *u* denotes the desired electromagnetic force, which is settled to obtain the driving currents. It consists of the output of the linear state error feedback controller (LSEF) *u*_0_ and the estimated observed total disturbance △*_ob_* obtained from the linearly expanding state observer (LESO). LSEF and LESO can be defined as:

$$\text{State space}:x_{1}=P_{i},x_{2}={\overset{\cdot }{P}}_{i},x_{3}=△,y=P_{i}$$ (15)

$$\text{LSEF}:u_{0}=k_{p}\left(\mathrm{ref}-{\hat{x}}_{1}\right)+k_{i}\left(\overset{\cdot }{\mathrm{ref}}-{\hat{x}}_{2}\right)$$ (16)

$$\begin{array}{c} \text{LESO}:\left[\begin{array}{c} {\overset{\cdot }{\hat{x}}}_{1}\\ {\overset{\cdot }{\hat{x}}}_{2}\\ {\overset{\cdot }{\hat{x}}}_{3} \end{array}\right]=\left[\begin{array}{ccc} -\beta_{1} & 1 & 0\\ -\beta_{2} & 0 & \frac{1}{m}\\ -\beta_{3} & 0 & 0 \end{array}\right]\left[\begin{array}{c} {\hat{x}}_{1}\\ {\hat{x}}_{2}\\ {\hat{x}}_{3} \end{array}\right]+u\left[\begin{array}{c} 0\\ \frac{1}{m}\\ 0 \end{array}\right]+y_{m}\left[\begin{array}{c} \beta_{1}\\ \beta_{2}\\ \beta_{3} \end{array}\right] \end{array}$$ (17)

The total disturbance is defined as an extended state in Eq. 15. LSEF is expressed in Eq. 16, where *k_p_* and *k_i_* are the controller parameters. LESO is expressed as Eq. 17 and *β_j_* (*j* ∈ {1, 2, 3}) represent the observer gains to be chosen, while *y_m_* denotes the measured value of the system output. Due to the convergence of LESO (△*_ob_* → △), the electromagnetic driving system can be approximated as a second-order standard type as follows:

$$m\cdot {\ddot{p}}_{i}=u_{0}$$ (18)

The standard type of system mitigates uncertainties and nonlinearities compared with the original system. Therefore, LESO significantly enhances the system’s robustness by promptly observing and eliminating the total disturbance. The simulations of position control in the vertical direction have been implemented in MATLAB R2022b to investigate the control performances of the proposed ADRC controller. Reaching precise position control in the vertical direction presents a formidable challenge, as it necessitates compensating for the gravitational influence. Hence, it is an appropriate scenario to analyze the controller’s performance. Besides, the commonly utilized controller [i.e., the proportional-integral-derivative (PID) controller and the time-delay-estimation (TDE) controller] are employed as comparative methods to demonstrate the superiority of the proposed ADRC controller in this paper. The input signals of the step and sine wave in the vertical direction were employed in the simulation, with both signals having an amplitude of 5 mm and the sine wave having a period of 20 s. In addition, a disturbance was introduced in the form of a step signal with an amplitude equal to one-tenth of the maximum input force during the simulation. The rise time and settling time are utilized to characterize the rapidity and stability of the three controllers in the step response. Furthermore, a phase shift is employed to quantify the response speed of the controller in the sine wave response. Finally, the accuracy and robustness of the controllers are evaluated by the maximum positioning errors and root mean square error (RMS) errors. The maximum errors mentioned here specifically pertain to the positional errors that reach their peak following a disturbance, as shown in Fig. 3B. The step and sine wave response simulation results are illustrated in Fig. 3B and Table 2. The rise time and phase shift of the ADRC controller are 0.987s and −0.007°, respectively, suggesting that the proposed controller’s response resembles the PID controller and is considerably faster than the TDE controller. However, the ADRC controller demonstrates superior stability by achieving a 77.4% faster settle time than the PID controller. Meanwhile, it shows a significant advantage over the other two controllers regarding RMS error and maximum error. The RMS errors of the ADRC controller exhibit a reduction ranging from 16.7% to 63.3%, while the maximum errors induced by disturbances experience a decrease ranging from 57.1% to 83.0%. The timely observation of total disturbance by the ADRC controller effectively mitigates their impact and significantly enhances the accuracy and robustness of the system.

**Table 2.** Simulation results of different controllers

Assessment criteria	Step signal			Sine wave signal		
	TDE	PID	ADRC	TDE	PID	ADRC
Rise time (s)	3.477	0.826	0.987			
Settling time (s)	7.980	5.854	1.323			
Phase shift (°)				−0.066	−0.024	−0.007
RMS error (μm)	753.9	480.8	400.6	828.8	517.0	189.5
Maximum error (μm)	585.7	413.0	99.5	3,141.4	2,398.1	1,029.1

### Implementation of a virtual boundary for enhanced interactive security via collision avoidance

The virtual boundary has been implemented to prevent any interferences or accidental collisions during operation, which enhances the security of the electromagnetic driving system. The proposed ADRC controller with high robustness can effectively mitigate most interferences within the constraint range. However, there remains a possibility of unexpected interferences when the micro-robot is exposed to strong external disturbances near the constraint range. The inadvertent interferences occurring in close proximity to the boundaries of the constraint range necessitate effective circumvention. Given the potential for catastrophic consequences in delicate procedures, such as intraoperative and postoperative complications, it is imperative to address this issue with utmost caution. Hence, the virtual binding force *F_v_* achieved by the controlled magnetic force of the electromagnetic driving system is utilized to provide a protective effect similar to medical gels, as illustrated in Fig. 3C. It can be defined as:

$$\left\{\begin{array}{c} F_{v}=-\frac{\mathrm{sgn}\left(\left\|P\right\|-l_{b}\right)+1}{2}\cdot \left(F_{\mathrm{vs}}+F_{\mathrm{vd}}\right)\\ F_{\mathrm{vs}}=k_{v}\cdot \frac{P}{\left\|P\right\|}\cdot \left(\left\|P\right\|-l_{b}\right)\\ F_{\mathrm{vd}}=b_{v}\cdot \left(\mathrm{sgn}\left(\frac{d\left\|P\right\|}{dt}\right)+1\right)\cdot \frac{\overset{\cdot }{P}}{2} \end{array}\right.$$ (19)

where *l_b_* denotes the radius of the virtual boundary. ***F***_***vs***_ and ***F***_***vd***_ represent the force vectors generated by the virtual spring and virtual damping, respectively; while *k_v_* and *b_v_* are spring and damping parameters of the virtual boundary, respectively. The simulation response to the sine wave input signal with persistent disturbances is also generated in MATLAB R2022b to validate the feasibility and effectiveness of the proposed virtual boundary scheme. The sinusoidal signal is subjected to disturbances introduced at the peaks or troughs, and the radius of the safety and virtual boundary was set to 2.25 mm and 2 mm, respectively. The simulation results are depicted in Fig. 3D, illustrating that the micro-robot surpasses the safety boundaries under disturbances in the absence of a virtual boundary. However, introducing the virtual boundary not only ensures constant confinement of the micro-robot within the electromagnetic workspace but also mitigates disturbance-induced fluctuations. These simulation results validate the efficacy of the proposed virtual boundary in preventing inadvertent collisions among micro-robots near the boundary, thereby enhancing the security of the designed system.

## Experiments and Results

### Experimental setup

**Fig. 4.**
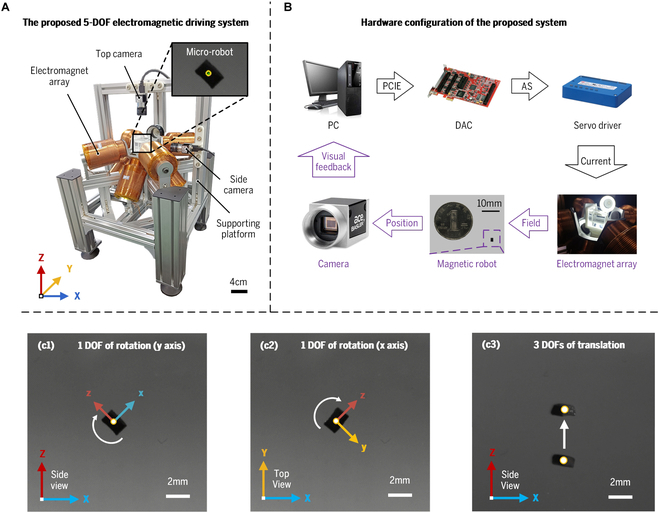
(A) Experimental setup for characterizing the performances of the proposed 5-DOF electromagnetic driving system. (B) Hardware configuration of the proposed system. (C) Micro-robot performing 5-DOF motion under magnetic manipulation.

The experimental setup has been established to characterize the performances of the proposed 5-DOF electromagnetic driving system, as illustrated in Fig. 4A and B. It primarily consists of an electromagnet array with eight optimized electromagnets, a supporting platform composed of aluminum profiles, a customized micro-robot with dimensions of 2 mm × 1 mm × 1 mm, two industrial cameras, a host computer, a data acquisition card (DAC) with eight channels, and four servo drivers with two channels each. Each electromagnet in the array is assembled on the supporting platform in the calculated optimal pose configuration. This electromagnet array can manipulate the micro-robot into 3-DOF translation in any direction and 2-DOF rotation motions except for rotating around its axis of the magnetic moment (represented by the *z* axis), as shown in Fig. 4C. The micro-robot is made of rubber magnets and is placed in a clear cubic container with a side length of 5 cm within the center of the electromagnet array. The clear cubic container provides a liquid environment for the micro-robot, and the surrounding fluid is silicone oil with a dynamic viscosity of 1.197 Pa·s, similar to the practical ophthalmic surgery scenarios. The overall workflow is shown in Fig. 4B. The host computer runs control and trajectory planning programs and transmits control signals to the DAC via the PCIE bus. The DAC (Sensoray 826) generates the analog signals amplified by the servo drivers (Copley AE2-090-30) to power the electromagnets. Two industrial cameras (Basler acA1440-220uc) are installed on the support platform to capture images of the micro-robot in real time from the top and side views and provide positional feedback for the proposed 5-DOF electromagnetic system.

**Fig. 5.**
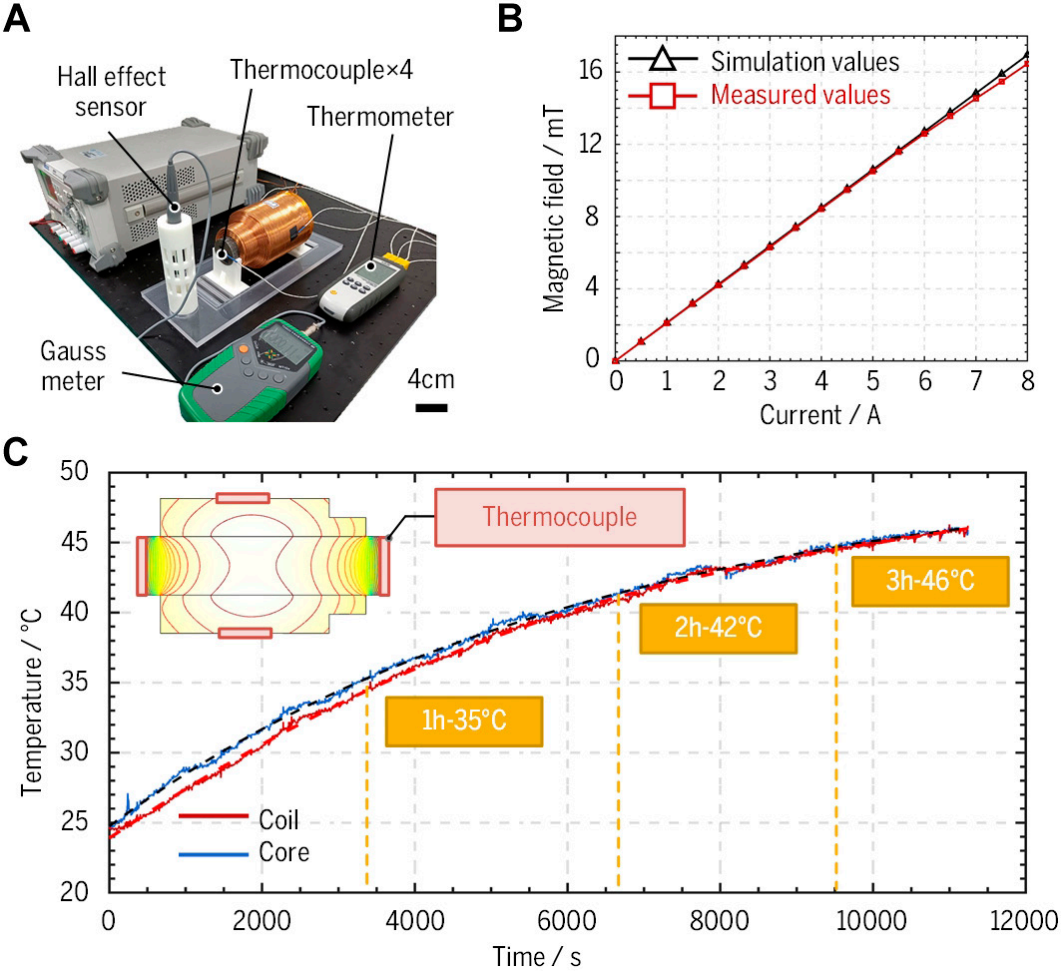
Magnetic field generation capability and thermal power performance tests of the optimized electromagnet. (A) Experimental setup. (B) Linear relationship between the applied current and the magnetic field. (C) Fluctuation in coil and core temperature over a 3-h period.

### Verification for magnetic field generation capability and thermal power of a single optimized electromagnet

The hardware configuration for measuring magnetic flux density and temperature has been established, as shown in Fig. 5A. It consists of a Gaussmeter, a Hall effect sensor, a thermometer, and four thermocouples. The Hall effect sensor measures the magnetic flux density generated by the optimized electromagnet, and the value is displayed on the Gaussmeter. The thermal field maps resulting from electromagnet energization were obtained through COMSOL software simulation, revealing the locations of maximum temperatures on coil and iron core surfaces, as illustrated in Fig. 5C. The maximum temperatures characterizing the optimized electromagnet’s thermal performance were measured by four thermocouples attached to corresponding positions and recorded utilizing a thermometer.

The DC power supply drives the electromagnet with an interval step of 0.5 A to generate a magnetic field within the range of [0, 8 A]. The measured and simulated magnetic flux density values are compared, as depicted in Fig. 5B. The mean error between them is −0.122 mT with an RMS error of 0.185 mT, thereby substantiating the validity of the proposed design optimization. The linear fitting method was used to fit the measured value to determine the relationship between magnetic flux density and current, and the coefficient of determination is 99.98%, indicating that the linearity of the electromagnet is excellent. The fitting results show that the proposed electromagnet with optimized parameters can generate a magnetic field of 2.071 mT at a distance of 65 mm with a current of 1 A. Compared with the standard 5-DOF electromagnetic driving system OctoMag, the magnetic field generation capability of the optimized electromagnet improved by approximately 33%, providing strong power support for the micro-robot during magnetic manipulation. However, the error between the measured and simulated magnetic flux density values appears to escalate with increasing current, potentially due to the near-saturation magnetization of the magnetic field generated by the iron core.

Moreover, the thermal performance of the optimized electromagnet has been verified at room temperature (25 °C) under natural heat dissipation. The supplied current is chosen as 2.5 A in these tests, producing a magnetic field sufficient to cover most magnetic manipulation scenarios. The experimental results show that the electromagnet’s temperature only increased from room temperature to a limited temperature of 46 °C in a relatively prolonged duration of 3 h, as depicted in Fig. 5C. This time duration is proper to perform the relevant surgical operations. This result indicates the superior comprehensive performance of the optimized electromagnet, facilitating longer-term magnetic manipulation. In summary, the optimized electromagnet has shown excellent comprehensive performances in the experimental tests. This proves that the proposed multi-objective design optimization method achieves a proper trade-off between magnetic field generation capability and thermal power.

### 3D simple trajectory tracking performance tests in the automatic operation mode

**Fig. 6.**
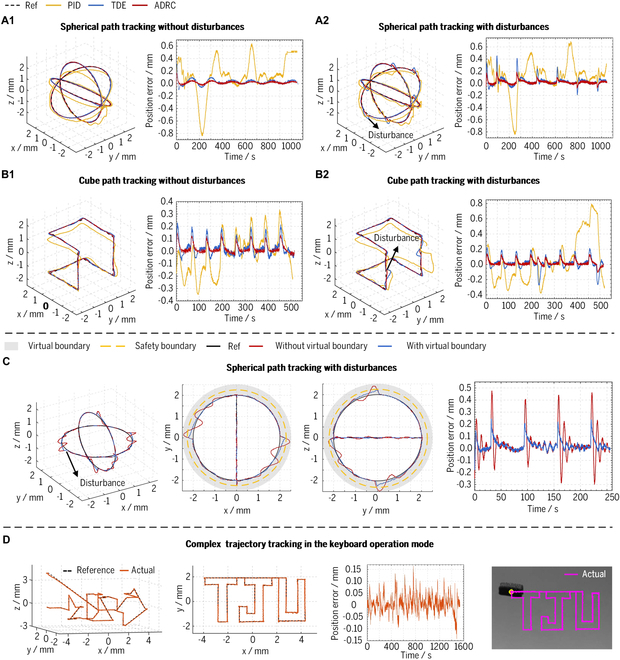
Experiments on 3D trajectory tracking. (A) Spherical path tracking results in automatic operation mode. (B) Cube path tracking results in automatic operation mode. (C) Spherical path tracking with virtual boundary enabled. (D) Complex trajectory tracking results in the keyboard operation mode.

Trajectory tracking experiments in the automatic operation mode were conducted on a spherical path (with a diameter of 4 mm) and a cube path (with a side length of 3 mm) to assess the accuracy and robustness of the proposed system, as depicted in Fig. 6A and B. The micro-robot tracked the reference paths divided into several discrete points, and the sampling frequency was approximately 30 Hz during experiments. The presented ADRC control method was utilized to solve the actuation currents and manipulate the micro-robot. Two other typical model-free controllers (PID and TDE) have also been conducted to compare with the proposed approach. Furthermore, the disturbances using electromagnetic forces were added to the path tracking process to validate the robustness of the proposed control method. The disturbances are around one-tenth of the maximum input force, orientated perpendicular to the current direction of motion, and were continuously fed into the system when the micro-robot moved to the specified position.

The RMS errors and maximum errors for path tracking experiments have been calculated, as presented in Table 3. The recorded data demonstrate that the ADRC control method yields the lowest error for both path types in the undisturbed case. The RMS error values were below 33.6 μm, while the maximum errors remained below 169.4 μm, indicating a significant improvement of 39.2% to 56.5% compared to TDE and 73.1% to 90.4% compared to PID controllers. The proposed 5-DOF electromagnetic driving system demonstrates superior spatial trajectory tracking accuracy when employing the ADRC control method, owing to its effective compensation for gravitational and fluid resistance forces. In particular, the ADRC control method exhibits the lowest maximum and RMS errors when disturbances are applied during path tracking, as listed in Table 3. The maximum errors and RMS errors observed after introducing disturbances indicate the controller’s robustness to a certain extent. Hence, the robustness of the electromagnetic driving system increased by approximately 47.4% to 88.2% in the case of the ADRC control method compared with the other two controllers. The ADRC control method is demonstrated to enhance the robustness of the proposed 5-DOF electromagnetic driving system.

**Table 3.** Position error during the simple trajectory tracking experiments

Position error		TDE		PID		ADRC	
		Cube	Sphere	Cube	Sphere	Cube	Sphere
RMS error	Without disturbances	57.0 μm	35.9 μm	177.1 μm	162.2 μm	33.6 μm	15.6 μm
	With disturbances	68.1 μm	49.1 μm	303.2 μm	171.9 μm	35.8 μm	21.9 μm
Maximum error	Without disturbances	278.7 μm	301.3 μm	629.2 μm	674.9 μm	169.4 μm	155.4 μm
	With disturbances	424.1 μm	363.1 μm	868.9 μm	679.4 μm	172.2 μm	159.3 μm

### Verification of virtual boundary for system security enhancement

The spherical path (with a radius of 2 mm) tracking experiments with disturbances were conducted to verify system security enhancement with virtual boundary, as depicted in Fig. 6C. The experiment assumes a virtual boundary in the form of a sphere with a radius of 2 mm, smaller than the safety boundary radius of 2.25 mm. Additionally, the disturbances applied are around two-fifths of the maximum input force. When virtual boundaries are not enabled, the maximum error for the path tracking experiment is 247.6 μm, which indicates that the micro-robot has exceeded the boundary by 22.6 μm in its motion. The maximum error can be reduced to 224.3 μm, decreasing by 9.4% with the virtual boundary enabled. It indicates that the micro-robot has not exceeded the boundaries within the constraints of the virtual boundary and has always maintained a distance greater than 0.7 μm during its motion. In summary, the virtual boundary effectively avoids the micro-robot being disturbed and thus colliding with the boundary during its movement, significantly improving the security of the proposed electromagnetic driving system.

### 3D complex trajectory tracking performance tests in the keyboard operation mode

The keyboard operation mode has been used to simulate micro-manipulation scenarios with complex trajectory-tracking experiments during intraocular surgery. The manipulator operated the micro-robot to track the 3D “TJU” trajectory through the keyboard. The comparison results between the measured and reference path are presented in Fig. 6D. The corresponding maximum error and RMS error values are 164.8 and 35.4 μm, respectively. The experimental results demonstrate that the proposed 5-DOF electromagnetic driving system can accurately track complex trajectories in the keyboard operation mode.

## Discussion

This work focuses on a novel 5-DOF electromagnetic driving system and its control framework based on the ADRC controller and virtual boundary. The primary challenge in scaling up magnetic driving systems to practical application sizes lies in their limited ability to generate high-intensity magnetic fields within a large workspace. Reinforcing the system’s power may alleviate such issues; however, this could potentially sacrifice operational sustainability due to defective thermal performance. The presented two-step design optimization method has demonstrated its effectiveness in providing strong sustained work capacity, overcoming the above issue. This approach increases the weight of force generation capacity in the vertical direction to reduce additional power consumption due to gravity compensation. Besides, the magnetic field-to-thermal power ratio is utilized as the objective function, which encompasses the comprehensive performance of the electromagnet while mitigating the influence of current magnitude. Therefore, the utilized design optimization significantly enhances the sustained work capacity of the proposed system. In addition, high compatibility with other ophthalmic operating instruments is also achieved during this optimization by allocating space above the workspace. Moreover, this system achieved a relatively compact configuration through electromagnet optimization targeting system volume, thereby minimizing space occupation and facilitating its application in compact surgical rooms and seamless coordination across various surgical areas. The performance evaluation was implemented with the optimized electromagnet and validated its performance and effectiveness. The optimal parameters of the electromagnet result in a strong magnetic field generation capability of 2.071 mT at a distance of 65 mm when energized with a current of 1 A. Furthermore, the electromagnet demonstrates minimal temperature elevation from room temperature (25 °C) to 46 °C through natural heat dissipation in 3 h, thereby facilitating sustained performance maintenance during prolonged and continuous operation. This limited temperature increase of the proposed micro-system poses negligible risk to the patient’s eyeball during the procedure, which can be elucidated by considering two key aspects. On the one hand, the measured temperature of 46 °C represents the surface temperature of the electromagnet after 3 h of continuous operation. However, it is important to note that the electromagnets do not come into direct contact with the patient’s eyeball. Therefore, the patient’s eyeball is supposed to present a lower temperature compared to the measured temperature due to the presence of air. On the other hand, temperatures of 50 °C or higher during surgical procedures may cause corneal burns [32]. This demonstrates that the operating temperature of the system remains within safe limits. Furthermore, future work will also explore implementing fiber Bragg grating (FBG)-based real-time detection of electromagnet temperatures, aiming to enhance safety measures [33,34].

Besides, the performance of the ADRC controller incorporating gravity compensation is investigated in this work to ensure consistency of motion performance in all directions. The virtual boundary offers a protective effect similar to medical gels, effectively mitigating interaction risk and further enhancing system security. The real-time trajectory tracking experiments have been conducted in different operation modes to provide empirical evidence for the effectiveness of the proposed control framework. Meanwhile, the comparison results with other related works [19,26,30,35,36] have been summarized and listed in Table 4. The results indicate a significant decrease in both the maximum error and maximum RMS error during disturbance-free performance tests, with reductions ranging from 47.1% to 65.4% and 62.7% to 84.4%, respectively. Besides, the performance tests conducted in this work have additionally taken into account disturbances that were overlooked by other related works. The obtained results demonstrate the system’s remarkable robustness in the presence of disturbances, as evidenced by the maximum error and RMS error being below 172.2 and 35.8 μm, respectively. In summary, the comparison results prove that the proposed control framework enables the electromagnetic driving system to effectively manipulate a micro-robot with enhanced accuracy and stability. In addition, future work will employ a more accurate magnetic field–current model to further enhance the positioning accuracy and enhance usable workspace within the open volume [19,25,26,37,38].

**Table 4.** The performance comparison of several electromagnetic driving systems

Group	Maximum error		Maximum RMS error	
	Without disturbances	With disturbances	Without disturbances	With disturbances
Marino et al. [35]	490 μm	-	-	-
Li et al. [26]	≈350 μm	-	-	-
Xing et al. [19]	360 μm	-	-	-
Kim et al. [36]	472 μm	-	215 μm	-
Fan et al. [30]	320 μm	-	90 μm	-
This work	169.4 μm	172.2 μm	33.6 μm	35.8 μm

## Conclusion

A novel electromagnetic driving system consisting of eight optimized electromagnets arranged in an optimal configuration is proposed in this work while utilizing a control framework based on the ADRC controller and virtual boundary. The presented system offers advantages in terms of high compatibility with other instruments, superior comprehensive performance on magnetic field generation capacity and thermal power, stronger capacity for sustained work, compact platform volume, superior accuracy and stability, and excellent robustness. Moreover, the employed two-step design optimization provides a guideline for performance improvement of the system configuration and electromagnet parameters. Besides, implementing the ADRC controller can significantly enhance the system’s robustness and security by effectively monitoring and compensating for total disturbances. Meanwhile, the realization of virtual boundaries enhances interactive security by facilitating collision avoidance. Hence, the proposed system is more suitably designed for performing intricate magnetic manipulation within the posterior eye during intraocular microsurgery. Characterization experiments have been conducted to evaluate the performances of the proposed system in terms of magnetic field generation capacity, thermal performance under natural heat dissipation, and trajectory tracking performance with disturbances. Future work will further consider the robotization and integration of additional instruments for the micro-robot’s injection with the proposed 5-DOF electromagnetic driving system to form a milli-micro combination ophthalmic surgical system.

## Data Availability

The data that support the findings of this study are available from the corresponding author upon reasonable request.
